# The Functional Spatio-Temporal Statistical Model with Application to O_3_ Pollution in Beijing, China

**DOI:** 10.3390/ijerph17093172

**Published:** 2020-05-02

**Authors:** Yaqiong Wang, Ke Xu, Shaomin Li

**Affiliations:** 1 Guanghua School of Management, Peking University, Beijing 100871, China; yaqiongwang@pku.edu.cn (Y.W.); lsmjim@pku.edu.cn (S.L.); 2 School of Statistics, University of International Business and Economics, Beijing 100029, China

**Keywords:** spatio-temporal statistical model, functional data analysis, O_3_ pollution

## Abstract

In recent years, with rapid industrialization and massive energy consumption, ground-level ozone ($O_{3}$) has become one of the most severe air pollutants. In this paper, we propose a functional spatio-temporal statistical model to analyze air quality data. Firstly, since the pollutant data from the monitoring network usually have a strong spatial and temporal correlation, the spatio-temporal statistical model is a reasonable method to reveal spatial correlation structure and temporal dynamic mechanism in data. Secondly, effects from the covariates are introduced to explore the formation mechanism of ozone pollution. Thirdly, considering the obvious diurnal pattern of ozone data, we explore the diurnal cycle of $O_{3}$ pollution using the functional data analysis approach. The spatio-temporal model shows great applicational potential by comparison with other models. With application to $O_{3}$ pollution data of 36 stations in Beijing, China, we give explanations of the covariate effects on ozone pollution, such as other pollutants and meteorological variables, and meanwhile we discuss the diurnal cycle of ozone pollution.

## 1. Introduction

As one of the major pollutants, ground-level ozone ($O_{3}$) has received a lot of public attention. Lots of studies have shown that $O_{3}$ could have detrimental effects on human health, including exacerbation of cardiovascular and respiratory dysfunction, and even premature mortality [1,2]. Additionally, tropospheric ozone, as a greenhouse gas, plays an important role in climate change, and further affects, for example, agricultural crop production [3,4]. In recent years, as the consequence of rapid industrialization and alarmingly increasing energy consumption, China has encountered severe air pollution [5,6,7,8]. Particularly, ozone becomes one of the serious and worsening pollutants in major areas of China, such as Beijing–Tianjin–Hebei urban agglomeration, and the Pearl River delta [9,10]. With a population of over 20 million, Beijing is one of the world’s largest mega cities. Due to coal burning, fugitive dust, and more recently a rapid increase in vehicular emissions, Beijing faces serious air pollution problems, and especially, studies regarding photochemical ozone pollution are attracting more and more attention [11,12].

The Chinese government identifies the urgency for air quality assessment and emission control, and has built a large monitoring network since 2013. Now, there are over 1500 national pollution monitoring stations in over 300 cities. Hourly readings of air pollutants are regularly recorded and directly transferred to China National Environmental Monitoring Center (CNEMC). The real-time observation and recording of the air pollution data provide a solid basis for studying the dynamic changes of pollutants and the underlying causes. Air quality data are collected over space and time; thus, the amount of data are large, and the analysis is complex. One important and common statistical characteristic of such data worthy of our notice is that the nearby (both in space and time) observations tend to be more alike than those far apart. Consequently, an assumption that spatio-temporal data follows the “independent and identically distributed” (iid) statistical paradigm should typically be avoided. Based on the underlying spatio-temporal structure of the pollution data, spatio-temporal statistical model, which simultaneously considers both the spatial covariance and temporal dependence, is thus a sensible and reasonable choice [13]. Moreover, $O_{3}$ data show a clear diurnal cycle. It peaks during the day and reaches a minimum at night. Since ozone data are sampled at a high frequency in time, it provides an overview of the daily cycle of pollutant concentrations.

A spatio-temporal statistical model is powerful to reveal spatial correlation structure and temporal dynamic mechanism in data. Huang and Cressie (1996) [14] introduced a dynamic random field with a separable spatio-temporal covariance structure, which is widely used in the environmental field. When the spatio-temporal dependencies become complicated, the power of the hierarchical statistical modeling (HM), which is capable of decomposing an uncertainty source of data, becomes apparent. The HM’s strength is well discussed in Cressie et al. [15]. Moreover, the daily pattern of ozone pollution needs more exploration. To do this, we divided the collection time into two parts, one related to intra-day fluctuations and the other related to intra-day changes. Geographic space is defined by latitude and longitude, with the date being the third dimension, and the intra-day hour is regarded as the fourth dimension, which gives a four-dimensional representation of the data. In this way, the functional data analysis (FDA) approach [16] is used to model the intra-day variation of the measurement data, and the remaining dimensions are processed according to the classic spatio-temporal data modeling. To summarize, in addition to the dynamic random field and the hierarchical modeling, the third building block is based on the functional representation of daily profiles of atmospheric pollution through a functional data analysis approach, which is the main innovation of the method.

In the present study, we propose a functional spatio-temporal statistical model, which is also a two-level hierarchical spatio-temporal model. A fruitful approach is based on the representation of random functional objects as linear combinations of the basis functions with Gaussian random coefficients. This allows for representing a functional model as a random components model and inheriting the related inferential machinery, e.g., Wood [17]. Based on the Kalman filter and expectation–maximization (EM) algorithm, a model inference for parameter estimates is implemented [18,19]. In addition, from the marginal likelihood function, an information matrix is obtained to measure the uncertainty of the model parameters [20]. The proposed model has the following advantages: (i) the dynamic random field is used to describe the spatio-temporal characterization of emissions of air pollution; (ii) and covariate effects are incorporated to analyze the underlying formation mechanism of atmospheric pollutants; (iii) in addition, the main innovation is the introduction of the functional data analysis approach, which is performed to explore the daily pattern of pollutants. In the paper, we show the capability of the model by using $O_{3}$ pollution data from 36 pollution monitoring stations in Beijing, China.

The paper is organized as follows. In Section 2, we describe the data in the study region, and introduce the Fourier basis functions, and the functional spatio-temporal statistical model, including the implementation of model estimation and cross-validation. In Section 3, we first show the selection of covariates and basis numbers. After comparing our model with others, we show the outstanding model capability, and finally give a comprehensive interpretation of the results. Conclusions are in Section 4.

## 2. Material and Methods

In this section, we first describe the data in the study region. Then, we introduce the Fourier basis function, and describe the functional spatio-temporal statistical model. In particular, model equations, model estimation, and cross-validation are discussed.

### 2.1. Data Description

The World Health Organization (WHO) set a guideline of 100 μg/m${}^{3}$ for a maximum daily 8-h average exposure to ground-level $O_{3}$; otherwise, adverse impacts on human health may occur [21]. Considering the increasing public concern on ozone, we attempt to analyze the effects from other pollutants and meteorological variables on ozone pollution, and provide some insight into the diurnal cycle of $O_{3}$, which peaks in the mid-day and reaches minimum at night-time.

In this study, we collect hourly concentration of the ground-level ozone in spring, summer, and autumn of year 2017, from thirty-six pollution monitoring stations in Beijing, China, which are directly managed by the Ministry of Environment and Protection (MEP). We also collect four other pollutant gases—particulate matter (${\mathrm{PM}}_{10}$), sulfur dioxide (${\mathrm{SO}}_{2}$), nitrogen dioxide (${\mathrm{NO}}_{2}$), and carbon monoxide (CO). All of the pollutant gases are measured in μg/m${}^{3}$. The oxides of nitrogen (${\mathrm{NO}}_{x}$) and the volatile organic components (VOC) constitute are known to be the important precursors of the ground ozone generation [22]. However, components of VOC are not measured by the air quality monitoring network.

**Figure 1:**
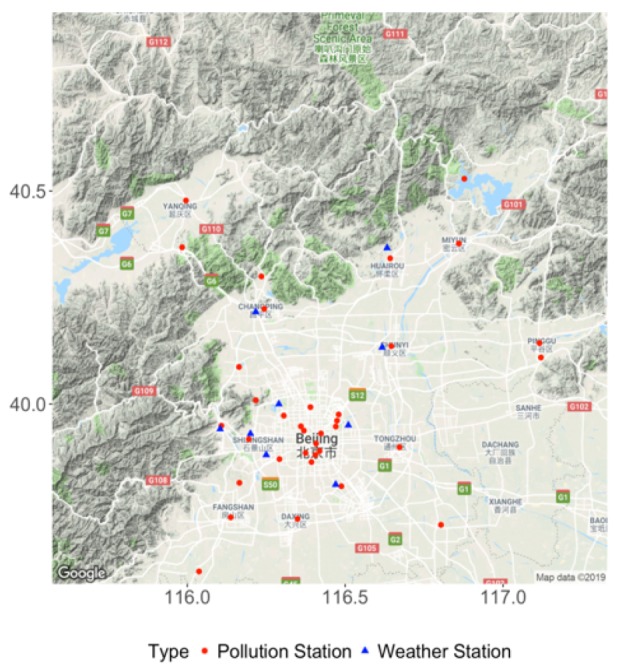
Thirty-six air quality monitoring stations with red dots and nine meteorological stations with blue triangles.

We also collect meteorological data: barometric pressure (PRES, in hectopascal), air temperature (TEMP, in degree celsius), dew point temperature (DEWP, in degree celsius), integrated rainfall (IRAIN, in millimeter), and integrated wind speed (Iws, in meter per second) from nine weather stations of China Meteorological Administration (CMA). All the measurements are recorded hourly. We match between air quality stations and meteorological stations by the geodesic distance. Figure 1 displays the spatial locations of the air quality stations with red dots as well as the meteorological stations with blue triangles [23]. In addition to these meteorological variables, ultraviolet radiation is also a significant meteorological factor that influences $O_{3}$ generation. Therefore, we download the data of UVB (in J/m${}^{2}$) with wavelengths between 200 and 440 nanometers from the European Centre for Medium-Range Weather Forecasts (ECMWF, https://cds.climate.copernicus.eu). The UVB data are provided at a grid size of 0.25°×0.25° at hourly frequency available over the study region. Since the UVB data vary greatly during the day and night, we take their log-transform before adding to the model. Note that the integrated rainfall and integrated wind speed are respectively calculated by:

(1) $$\begin{array}{c} \begin{array}{c} IWS_{t}=\left\{\begin{array}{cc} WS_{t}, & WD_{t}!=WD_{t-1},\\ IWS_{t-1}+WS_{t}, & WD_{t}==WD_{t-1}. \end{array}\right. \end{array} \end{array}$$

(2) $$\begin{array}{c} \begin{array}{c} IRAIN_{t}=\left\{\begin{array}{cc} RAIN_{t}, & RAIN_{t}=0,\\ IRAIN_{t-1}+RAIN_{t}, & RAIN_{t}!=0. \end{array}\right. \end{array} \end{array}$$

### 2.2. Fourier Basis

The basic philosophy of functional data analysis is to think of observed data functions as single entities, rather than merely as a sequence of individual observations. In practice, functional data are usually observed and recorded discretely as *n* pairs $\left(t_{j},y_{j}\right)$, and $y_{j}$ is a snapshot of the function at time $t_{j}$, possibly blurred by measurement error. Time is so often the continuum over which functional data are recorded that we may slip into the habit of referring to $t_{j}$ as such, but certainly other continua may be involved, such as spatial position, frequency, weight, and so forth:

(3) $$\begin{array}{c} y_{j}=x\left(t_{j}\right)+\epsilon_{j} \end{array}$$

In functional data analysis, we need a strategy for constructing functions, which balances the model fitting and complexity. We built a set of functions where $\phi_{k},k=1,\ldots ,K$ are called basis functions, and their linear combination is defined as a function:

(4) $$\begin{array}{c} x\left(t\right)=\sum_{k=1}^{K}c_{k}\phi_{k}\left(t\right)=c^{\prime }\phi \left(t\right), \end{array}$$

the expansion of the basis function, where the parameters $c_{k},k=1,\ldots ,K$ are the expansion coefficients to be estimated. In effect, basis expansion methods represent the potentially infinite dimensional world of functions within the finite-dimensional framework of vectors like c. The functional data analysis is simplified to multivariate data analysis.

**Figure 2:**
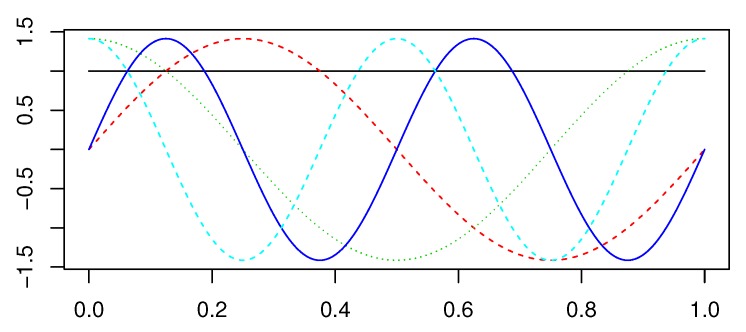
Fourier basis function system with K=5 and T=1.

**Figure 3:**
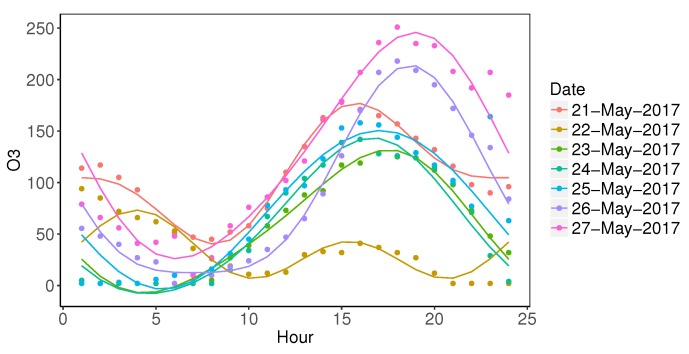
Ozone data fitting by using five Fourier basis functions.

The basis functions used for data modeling mostly belong to two categories: periodic and non-periodic. Most functional data analyses involve either a Fourier basis for periodic data, or a B-spline basis for non-periodic data. Since we are interested in the diurnal variations of ozone, we introduce the Fourier basis functions in detail. In order to express the repeated pattern in long-term sequences, basis functions need to be repeated within a certain time period *T*. The famous basis function extension for periodic data provided by the Fourier series is:

(5) $$\begin{array}{c} \hat{x}\left(t\right)=c_{0}+c_{1}sin\left(\omega t\right)+c_{2}cos\left(\omega t\right)+c_{3}sin\left(2\omega t\right)+c_{4}cos\left(2\omega t\right)+\ldots \end{array}$$

where ω=2π/T. Defining a Fourier basis system requires two pieces of information: the number of basis functions *K* and the period *T*. Figure 2 shows the Fourier basis system with K=5 and T=1. Followed by the constant, the Fourier basis functions are arranged in consecutive sine/cosine pairs:

We select the ozone data from one of the pollution stations—Wanliu Monitoring Station, which is located at Haidian District, Beijing, for preliminary analysis. The time span is one week from 21 May 2017 to 27 May 2017. We capture the daily variation of ozone data by using five Fourier basis functions. The mean square error (MSE) of fitted residuals is 14.79 μg/m${}^{3}$. As shown in Figure 3, the predicted value at hour 24 matches the predicted value at hour 0 in the next day, guaranteeing the periodic nature of the daily cycle.

### 2.3. Model Equation

Let $s=(s_{lat},s_{lon})$ be the generic spatial location on the Earth’s sphere with sample size *n*, and t=1,…,T the day index, and domain $H=\left[h_{1},h_{2}\right]\subset R$ the time within the day expressed in hours. The model for ozone observations $O_{3}\left(s,t,h\right)$ is:

(6) $$\begin{array}{cc} O_{3}\left(s,t,h\right) & =x{\left(s,t,h\right)}^{{}^{\prime }}\beta \left(h\right)+\phi {\left(h\right)}^{\prime }z\left(s,t\right)+\varepsilon \left(s,t,h\right), \end{array}$$

(7) $$\begin{array}{cc} z(s,t) & =Gz(s,t-1)+\eta (s,t). \end{array}$$

This model is referred to as the functional dynamic spatio-temporal model. In Equation (6), ε is a zero-mean Gaussian measurement error independent in space and time with functional variance $\sigma_{\varepsilon }^{2}\left(h\right)$, which implies that ε is heteroskedastic across the domain H. The variance is modeled as

(8) $$\begin{array}{c} \log\left(\sigma_{\varepsilon }^{2}\left(h\right)\right)=\phi {\left(h\right)}^{\prime }c_{\varepsilon }, \end{array}$$

where ϕ(h) is a p×1 vector of basis functions evaluated at *h* while $c_{\varepsilon }$ is a vector of coefficients to be estimated. In Equation (6), x(s,h,t) is a b×1 vector of covariates while $\beta \left(h\right)={\left(\beta_{1}\left(h\right),\ldots ,\beta_{b}\left(h\right)\right)}^{\prime }$ is the vector of functional parameters modeled as

(9) $$\begin{array}{c} \beta_{j}\left(h\right)=\phi {\left(h\right)}^{\prime }c_{\beta ,j}, \end{array}$$

and $c_{\beta }={\left(c_{\beta ,1}^{\prime },\ldots ,c_{\beta ,b}^{\prime }\right)}^{\prime }$ is the pb×1 vector of coefficients to be estimated. Additionally, z(s,t) is a p×1 latent space-time variable with Markovian dynamics given in Equation (7). Matrix G is a diagonal transition matrix with diagonal elements in the p×1 vector g. The vector η is described by a multivariate Gaussian process independent in time but correlated across space with matrix spatial covariance function given by

(10) $$\begin{array}{c} \Gamma \left(s,s^{\prime };\theta \right)=diag\left(v_{1}\rho \left(s,s^{\prime };\theta_{1}\right),\ldots ,v_{p}\rho \left(s,s^{\prime };\theta_{p}\right)\right), \end{array}$$

and $v={\left(v_{1},\ldots ,v_{p}\right)}^{\prime }$ is the vector of scale coefficients while $\rho (s,s^{\prime };\theta_{j})$ is a valid spatial correlation function for locations $s,s^{\prime }\in S^{2}$ parametrized by $\theta_{j}$, and $\theta ={\left(\theta_{1},\ldots ,\theta_{p}\right)}^{\prime }$. The unknown model parameter vector is $\psi =\left(c_{\varepsilon }^{\prime },c_{\beta }^{\prime },g^{\prime },v^{\prime },\theta^{\prime }\right)$.

**Figure 4:**
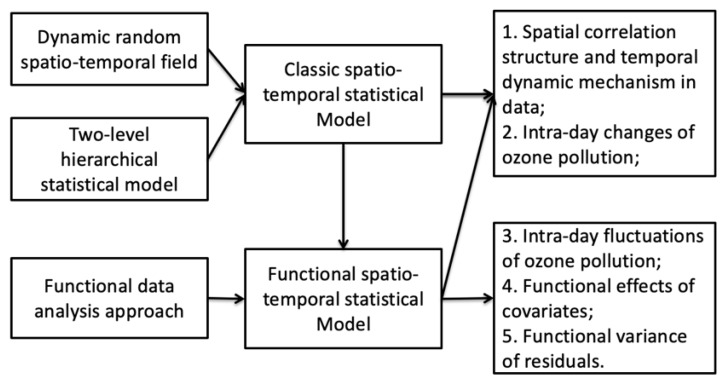
Methodology summary.

In Figure 4, we summarize the methodology. The main innovation is to incorporate the function data analysis approach to the classic spatio-temporal statistical model, which facilitates exploring the intra-day fluctuations of ozone pollution as well as the functional effects of covariates. Note that, in order to ease the notation, the same *p*-dimensional basis functions ϕ(h) are used to model $\sigma_{\varepsilon }^{2}$, $\beta_{j}$ and $\phi {\left(h\right)}^{\prime }z\left(s,t\right)$ in Equations (6) and (7). In the empirical analysis, we choose different numbers of basis functions for modeling according to the model criteria, such as the mean square error (MSE), and $R^{2}$ (see Section 2.5 for details).

### 2.4. Model Estimation

The estimation of ψ and the latent space-time variable z(s,t) is based on the maximum likelihood approach and Kalman filter. At a specific location $s_{i}$ and time *t*, *q* measurements are taken at hour points $h={\left(1,2,\ldots ,q\right)}^{\prime }$ and collected in the vector

(11) $$\begin{array}{c} y_{s_{i},t}={\left(O_{3}\left(s_{i},t,1\right),\ldots ,O_{3}\left(s_{i},t,q\right)\right)}^{\prime }, \end{array}$$

where q=24 as pollutants are hourly recorded. Daily profiles of ozone data observed at time *t* across spatial locations S are then stored in the vector $y_{t}={\left(y_{s_{1},t}^{\prime },\ldots ,y_{s_{n},t}^{\prime }\right)}^{\prime }$. Accordingly, Equations (6) and (7) are rewritten as

(12) $$\begin{array}{cc} y_{t} & ={\tilde{X}}_{t}c_{\beta }+\Phi_{z,t}z_{t}+\varepsilon_{t}, \end{array}$$

(13) $$\begin{array}{cc} z_{t} & =\tilde{G}z_{t-1}+\eta_{t}, \end{array}$$

where ${\tilde{X}}_{t}=X_{t}\Phi_{\beta ,t}$ is a nq×bp matrix, with $X_{t}$ the matrix of covariates and $\Phi_{\beta ,t}$ the basis matrix for β. $\Phi_{z,t}$ is the nq×np basis matrix for the latent np×1 vector $z_{t}={\left(z{\left(s_{1},t\right)}^{\prime },\ldots ,z{\left(s_{n},t\right)}^{\prime }\right)}^{\prime }$. $\eta_{t}={\left(\eta {\left(s_{1},t\right)}^{\prime },\ldots ,\eta {\left(s_{n},t\right)}^{\prime }\right)}^{\prime }$ is the np×1 innovation vector, while $\varepsilon_{t}$ is the nq×1 vector of measurement errors. Additionally, $\tilde{G}={G⊗I}_{n}$ is the np×np diagonal transition matrix.

The complete-data likelihood function L(ψ;Y,Z) can be written as

(14) $$\begin{array}{c} L\left(\psi ;Y,Z\right)=L\left(\psi_{z_{0}};z_{0}\right)\prod_{t=1}^{T}L\left(\psi_{y};y_{t}|z_{t}\right)L\left(\psi_{z};z_{t}|z_{t-1}\right), \end{array}$$

where $Y=\left(y_{1},\ldots ,y_{T}\right)$, $Z=\left(z_{0},z_{1},\ldots ,z_{T}\right)$, $\psi_{z}=\left(g^{\prime },v^{\prime },\theta^{\prime }\right)$, $\psi_{y}=\left(c_{\varepsilon }^{\prime },c_{\beta }^{\prime }\right)$, and $z_{0}$ is the Gaussian initial vector with parameter $\psi_{z_{0}}$. The model parameter set ψ is initialized with starting values $\psi^{\langle 0\rangle }$ and then updated at each iteration ι of the EM algorithm. The algorithm terminates if any of the conditions is satisfied

(15) $$\begin{array}{c} ∥\psi^{\langle \iota \rangle }-\psi^{\langle \iota -1\rangle }∥/∥\psi^{\langle \iota \rangle }∥<\epsilon , \end{array}$$

(16) $$\begin{array}{c} \left|L\left(\psi^{\langle \iota \rangle };Y\right)-L\left(\psi^{\langle \iota -1\rangle };Y\right)\right|/\left|L(\psi^{\langle \iota \rangle };Y)\right|<\epsilon , \end{array}$$

where   is the $l_{2}$ norm, $\psi^{\langle \iota \rangle }$ is the parameter set at the ι-th iteration, $L(\psi^{\langle \iota \rangle };Y)$ is the observed-data likelihood function evaluated at $\psi^{\langle \iota \rangle }$, and ϵ is a small positive number (e.g., $10^{-3}$).

The EM algorithm provides a point estimate of the parameter vector ψ but without uncertainty information. Note that Y is a vector with dimension N=nqT. Generally speaking, inverting the full variance–covariance matrix of the *N*-dimensional data vector Y has a computational complexity in the order of $O(N^{3})$, which is clearly unfeasible. Thanks to the state space representation of model, we estimate the variance–covariance matrix ${\hat{\Sigma }}_{\psi }=V\left(\psi |Y\right)$ from the marginal likelihood, which may be used for model selection and inference.

### 2.5. Cross-Validation

We implement a 2-fold cross-validation by partitioning the original spatial locations S into subsets $S_{est}$ and $S_{xval}$. Data related to $S_{est}$ are used for model estimation while data related to $S_{xval}$ are used for cross-validation. The cross-validation mean squared errors are then computed by

(17) $$\begin{array}{c} MSE_{s}=\frac{1}{B}\sum_{t=1}^{T}\sum_{h\in h_{s,t}}{\left(O_{3}\left(s,h,t\right)-\hat{O_{3}}\left(s,h,t\right)\right)}^{2}, \end{array}$$

where $\hat{O_{3}}\left(s,h,t\right)=E_{\hat{\phi }}\left(O_{3}\left(s,h,t\right)|Y\right)$ is the prediction of ozone data at the cross-validation stations, and *B* is the number of terms in each sum. We also obtain the cross-validation $R^{2}$ with respect to station s:

(18) $$\begin{array}{cc} R_{s}^{2} & =1-\frac{MSE_{s}}{VAR\left(\{O_{3}\left(s,h,t\right),t,h\}\right)} \end{array}$$

The choice of the numbers of basis functions is very essential for model estimation. Here, based on the cross-validated mean square error and other model criteria, we choose the reasonable numbers of basis functions to estimate $\sigma_{\varepsilon }^{2}$, $\beta_{j}$ and $\phi {\left(h\right)}^{\prime }z\left(s,t\right)$ respectively. After implementing leave-one-station-out cross-validation, we take the average $\bar{MSE}$ and $\bar{R^{2}}$ as our criteria:

(19) $$\begin{array}{c} \bar{MSE}=\frac{1}{n}\sum_{i=1}^{n}MSE_{s_{i}}, \end{array}$$

(20) $$\begin{array}{c} \bar{R^{2}}=\frac{1}{n}\sum_{i=1}^{n}R_{s_{i}}^{2}. \end{array}$$

## 3. Analysis of $O_{3}$ Pollution in Beijing

In the paragraph, we first show the selection of covariates and basis numbers with application to ozone data in Beijing, and then focus on the summertime modeling. By comparing our proposed model with other models, we show the outstanding advantage of the functional spatio-temporal statistical model. Finally, we show the model results and interpret the parameter estimates, especially the functional effects of covariates.

### 3.1. Selection of Covariates and Basis Numbers

In the following text, we select the covariates in x(s,t,h) by using the Akaike information criterion (AIC). Table 1 displays the results of forward selection based on AIC, which means starting with no covariates, and iteratively adding the most contributive covariates. For instance, in the summertime modeling, at the beginning (Iter 0), we select the variable ${\mathrm{NO}}_{2}$, which results in the best model performance with maximum AIC. Then, at the next iteration (Iter 1), the variable particulate matter (${\mathrm{PM}}_{10}$) is further selected. Table 1 shows that the importance of covariates varies among seasons, but the most important variables are ${\mathrm{SO}}_{2}$, ${\mathrm{NO}}_{2}$, and ${\mathrm{PM}}_{10}$.

**Figure 5:**
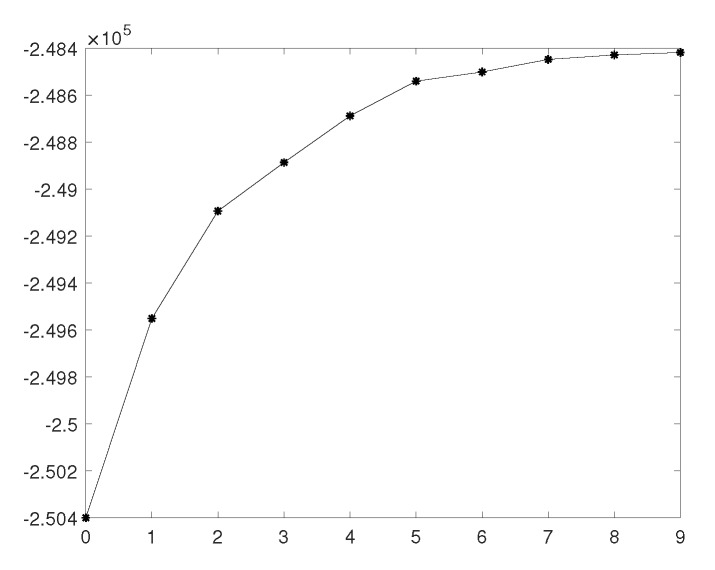
Improvement of AIC at each iteration for summertime modeling.

The ozone concentrations display a significant seasonal pattern, being pretty high in summer, while meanwhile being moderate in winter [24,25]. Therefore, we focus on the analysis of $O_{3}$ pollution in summer. Figure 5 shows the maximum AIC at each iteration for summertime modeling.

The improvement of model AIC is no longer significant after five iterations; therefore, we find the optimal subset of covariates—$NO_{2}$, $PM_{10}$, $SO_{2}$, TEMP, IRAIN, and UVB. Hence, the measurement equation for ozone data is

(21) $$\begin{array}{ccc} O_{3}\left(s,h,t\right) & = & \beta_{0}\left(h\right)+x_{NO2}\left(s,h,t\right)\beta_{NO2}\left(h\right)+x_{PM_{10}}\left(s,h,t\right)\beta_{PM_{10}}\left(h\right)\\ & & +x_{SO_{2}}\left(s,h,t\right)\beta_{SO_{2}}\left(h\right)+x_{TEMP}\left(s,h,t\right)\beta_{TEMP}\left(h\right)\\ & & +x_{IRAIN}\left(s,h,t\right)\beta_{IRAIN}\left(h\right)+x_{UVB}\left(s,h,t\right)\beta_{UVB}\left(h\right)\\ & & +\phi {\left(h\right)}^{\prime }z\left(s,t\right)+\varepsilon \left(s,h,t\right), \end{array}$$

where data are available at h=1,…,24, $s\in \{s_{1},\ldots ,s_{36}\}$, and t=1,…,92. Moreover, due to the circularity of time, Fourier basis functions are adopted. This implies that $\beta_{j}\left(h\right)$, $\sigma_{\varepsilon }^{2}\left(h\right)$ are periodic functions. Under the different combinations of the numbers of basis functions, the model criteria $\bar{MSE}$ and $\bar{R^{2}}$ in Section 2.5 are obtained and shown in Table 2.

From the table, when the number of basis functions for estimating $\phi {\left(h\right)}^{\prime }z\left(s,t\right)$ increases, it significantly reduces the $\bar{MSE}$. When the number of basis functions increases from 5 to 7, the $\bar{MSE}$ is reduced more than that from 7 to 9. Considering such enormous calculation stress, we choose seven basis functions to estimate the latent component $\phi {\left(h\right)}^{\prime }z\left(s,t\right)$. However, increasing the number of basis functions for the variance $\sigma_{\varepsilon }^{2}\left(h\right)$ of the residual ε(s,h,t) does not significantly reduce $\bar{MSE}$ but is helpful to improve the AIC. We find that an increase from 3 to 5 has an improvement in AIC, but the improvement becomes very minor from 5 to 7. Thus, we choose five basis functions to estimate the variance $\sigma_{\varepsilon }^{2}\left(h\right)$. Finally, we choose five basis functions to estimate the effects from covariates $\beta_{j}\left(h\right)$, considering the trade-off between the model interpretation and over-fitting problem. Based on the analysis above, the number of basis functions for $\beta_{j}\left(h\right)$, $\sigma_{\varepsilon }^{2}\left(h\right)$ and $\phi {\left(h\right)}^{\prime }z\left(s,t\right)$ is chosen to be 5, 5, and 7, respectively.

### 3.2. Model Comparison

In the paragraph, we compare the five models, namely Equations (22), (23), (24), (25), and (26). Equation (22) is an ordinary regression model; Equation (23) is a regression model with functional β(h) estimates; Equation (24) introduces the latent spatio-temporal variable z(s,t) to characterize the spatio-temporal correlation; Equation (25) is a simplified version of the proposed functional spatio-temporal statistical model that is β(h)≡β, $\sigma_{\varepsilon }^{2}\left(h\right)\equiv \sigma_{\varepsilon }^{2}$; Equation (26) is the functional spatio-temporal statistical model:

(22) $$\begin{array}{cc} O_{3} & =X\beta +\epsilon \end{array}$$

(23) $$\begin{array}{cc} O_{3}\left(h\right) & =X(h)\beta (h)+\epsilon (h) \end{array}$$

(24) $$\begin{array}{cc} O_{3}\left(s,t\right) & =X(s,t)\beta +z(s,t)+\epsilon (s,t)\\ z(s,t) & =Gz(s,t-1)+\eta (s,t) \end{array}$$

(25) $$\begin{array}{cc} O_{3}\left(s,h,t\right) & =X\left(s,h,t\right)\beta +\phi {\left(h\right)}^{\prime }z\left(s,t\right)+\epsilon \left(s,h,t\right)\\ z(s,t) & =Gz(s,t-1)+\eta (s,t) \end{array}$$

(26) $$\begin{array}{cc} O_{3}\left(s,h,t\right) & =X\left(s,h,t\right)\beta \left(h\right)+\phi {\left(h\right)}^{\prime }z\left(s,t\right)+\epsilon \left(s,h,t\right)\\ z(s,t) & =Gz(s,t-1)+\eta (s,t) \end{array}$$

Similar to the selection of the numbers of basis functions, the average $\bar{MSE}$ and $\bar{R^{2}}$, and AIC are used to assess the model performance. As shown in Table 3, our model Equation (26) is the optimal among the five models in view of the three model criteria. Equation (23) is much improved from Equation (22) in terms of $\bar{MSE}$ and $\bar{R^{2}}$, which means a better model forecast in general. Benefiting from the latent spatio-temporal variable z(s,t), Equation (24) has an unbeatable advantage over the ordinary regression models, accessing much smaller $\bar{MSE}$ and much larger $\bar{R^{2}}$ and AIC. Equation (25) introduces the functional data analysis approach, and characterizes the latent component as a linear combination of the basis functions and the latent random spatio-temporal variable z(s,t). Although the AIC is only a little increased, a smaller $\bar{MSE}$ and larger $\bar{R^{2}}$ are achieved. Eventually, when Equation (26) adds the functional covariate effects β(h) and the functional variance of the residuals $\sigma_{\epsilon }\left(h\right)$, $\bar{MSE}$, and $\bar{R^{2}}$ is not improved much. However, AIC is further improved, which benefits from the more capable interpretation of covariates and the flexibility of the residual variance.

**Figure 6:**
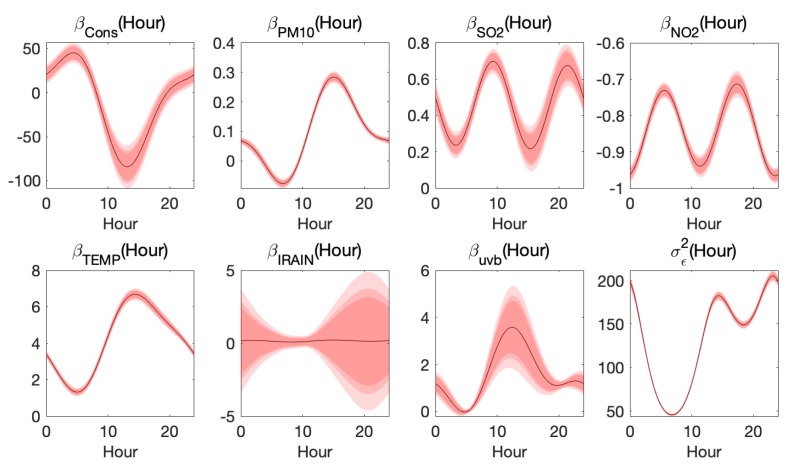
Estimated $\beta_{cons}\left(hour\right),\beta_{PM10}\left(hour\right),\beta_{SO2}\left(hour\right),\beta_{NO2}\left(hour\right),\beta_{TEMP}\left(hour\right),\beta_{IRAIN}\left(hour\right)$, $\beta_{UVB}\left(hour\right)$ and $\sigma_{\epsilon }^{2}\left(hour\right)$, with 90%,95%,and99%- confidence bands.

In Equation (26), firstly, the latent hidden variable z(s,t) captures the spatial correlation by range parameter θ, and variance parameter *v*, which shows that an average standard deviation of 48 μg/m${}^{3}$ of ozone data are explained by z(s,t) (refer to Table 5). Secondly, the functional β(h) shows that the covariate effects are both significant and nonlinear, indicating the complicated formation of ozone pollution by using the functional representation (refer to Figure 6). In summary, the hierarchical spatio-temporal statistical model, combined with functional data analysis approach, contributes to the high amount of $\bar{R^{2}}$.

### 3.3. Model Result

Figure 6 shows the estimated β(h) and $\sigma_{\epsilon }^{2}\left(h\right)$ for model Equation (21). Thanks to Fourier basis functions, the estimation result at the end of the day matches the beginning of the next day.Since, in general, the confidence bands of estimated β(h) may contain zero, it may be useful to test the significance of covariates. The $\chi^{2}$ tests are introduced as follows:

(27) $$\begin{array}{c} \beta_{j}\left(h\right)=\phi {\left(h\right)}^{\prime }c_{\beta ,j},\;c_{\beta ,j}∼N\left(0,\Sigma_{\hat{c_{\beta ,j}}}\right). \end{array}$$

Thus, $c_{\beta ,j}^{\prime }\Sigma_{\hat{c_{\beta ,j}}}^{-1}c_{\beta ,j}∼\chi^{2}\left(rank\left(\Sigma_{\hat{c_{\beta ,j}}}\right)\right)$. In Figure 6, IRAIN fluctuates around zero. The results of $\chi^{2}$ tests for the significance of covariates are reported in Table 4, and indicate that the effect of variable IRAIN is not jointly significant.

Therefore, it comes to the final model equation by excluding the IRAIN variable:

(28) $$\begin{array}{ccc} O_{3}\left(s,h,t\right) & = & \beta_{0}\left(h\right)+x_{NO2}\left(s,h,t\right)\beta_{NO2}\left(h\right)+x_{PM_{10}}\left(s,h,t\right)\beta_{PM_{10}}\left(h\right)\\ & & +x_{SO_{2}}\left(s,h,t\right)\beta_{SO_{2}}\left(h\right)+x_{TEMP}\left(s,h,t\right)\beta_{TEMP}\left(h\right)\\ & & +x_{UVB}\left(s,h,t\right)\beta_{UVB}\left(h\right)+\phi {\left(h\right)}^{\prime }z\left(s,t\right)+\varepsilon \left(s,h,t\right). \end{array}$$

In Table 5, we show the estimates and standard deviation of parameters relevant to the latent spatio-temporal variable z(s,t), which are the transition coefficient g, range parameter θ, and variance vector v. Most estimates of g parameter are positive, and the absolute values are all within one, which guarantees the stability of the 7-variate spatio-temporal process z(s,t). Compared with the geodesic distance of Beijing (around 50 km), the values of θ parameter, ranging from 31.92 km to 63.12 km, indicate a strong spatial correlation within the city. The average v estimate is around 2313 (with standard deviation of 48 μg/m${}^{3}$), and shows that the latent variable z(s,t) accounts for much more proportion of original $O_{3}$ variance than the unexplained term $\sigma_{\epsilon }^{2}\left(h\right)$. Hence, introducing the latent spatio-temporal variable z(s,t) guarantees the advantage of the proposed model.

**Figure 7:**
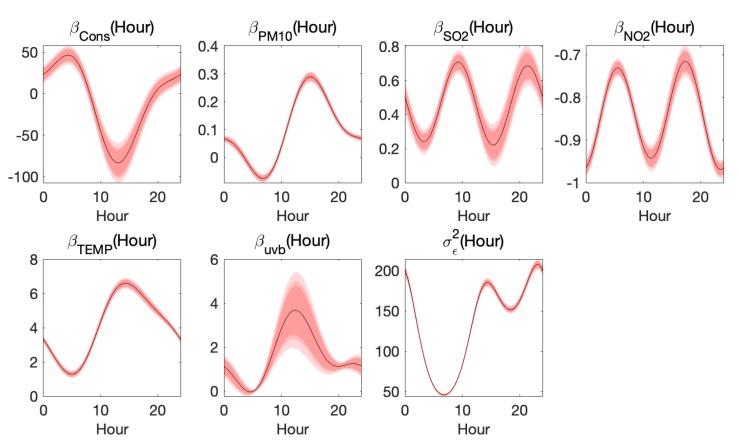
Estimated $\beta_{cons}\left(hour\right),\beta_{PM10}\left(hour\right),\beta_{SO2}\left(hour\right),\beta_{NO2}\left(hour\right),\beta_{TEMP}\left(hour\right),\beta_{UVB}\left(hour\right)$ and $\sigma_{\epsilon }^{2}\left(hour\right)$, with 90%,95%,and99%- confidence bands.

Finally, in Figure 7, we show the estimated β(h) and $\sigma_{\epsilon }^{2}\left(h\right)$. The last figure is the plot of functional variance $\sigma_{\epsilon }^{2}\left(h\right)$, which represents the unexplained portion of $O_{3}$ variance. The plot shows that the model is more capable when explaining the situation during the daytime [26].

As shown in Figure 7, the coefficient curves of TEMP, uvb and $PM_{10}$ are similar, increasing from early morning and attending the peak at 12:00 p.m.–2:00 p.m., then falling down. Focusing on daytime, we see that the three curves are consistent with the trend of temperature (or uvb), which implicates that the relationship between ozone and temperature (or uvb) might be quadratic [27], or there were interactions between temperature and uvb, that is, the ozone concentrations were dependent on $TEMP^{2}$, $uvb^{2}$, or TEMP×uvb. The coefficients of TEMP and uvb in daytime are positive, which is consistent with the present research [28]. While the coefficient of $PM_{10}$ is negative at 5:00 a.m.–10:00 a.m. and positive during other time periods. The positive correlation between $PM_{10}$ and ozone may be caused by their common sources, secondary nature, and interactions of their precursors [29], and the negative correlation could be explained by PM’s consumption of hydroperoxy (HO2) radicals, which would otherwise react with NO for ozone generation [30]. Furthermore, the positive correlation becomes the strongest at 3:00 p.m., at which time the ozone concentration attains the largest.

In addition, the coefficient curves of $NO_{2}$ and $SO_{2}$ both have two spikes, while the coefficient of $NO_{2}$ is negative and the other is positive. The negative relationship between $NO_{2}$ and ozone is consistent with results in many studies [31,32], and the positive correlation between $SO_{2}$ and ozone could be explained by their common dependences on meteorology [33]. The strongest correlation between $NO_{2}$ and ozone in daytime appears at about 11:00 a.m., and the weakest correlation appears at 5:00 a.m. and 6:00 p.m. In contrast, the correlation between $SO_{2}$ and ozone is the strongest at 9:00 a.m. and 8:00 p.m., in other words, approximately the end of morning/evening rush hours in Beijing, respectively, and such correlation is the weakest at 3:00 p.m.

## 4. Conclusions

In this paper, we propose a functional spatio-temporal statistical method to analyze air quality data, and explore the mechanism of pollution formation.

- The method has several advantages. First, as a hierarchical spatio-temporal statistical model, it is flexible enough to handle latent variable while capturing spatio-temporal dynamics. Second, the proposed model also takes covariates into consideration, thereby being efficient in discovering relational patterns from chemical reaction, and meteorological factors on the formation of $O_{3}$ pollution. Third, in the framework of the spatio-temporal models, we are the first to explore the intra-day variation of ozone through the functional data analysis approach, which is the most innovative part of the model.
- The model has made the following progresses. First of all, our model outperforms other models in many ways, as shown in Section 3.2. Second, the latent spatio-temporal variable z(s,t) well captures the temporal dynamic and spatial structure of ozone data. Third, from the functional effects of the covariates, we explore the possible effects of air pollutants and meteorological variables on ozone data.
- Our model is flexible enough to model any kind of data with spatio-temporal structure; therefore, it can be applied in many fields, such as economy and agriculture, apart from the environment. The introduction of the functional data analysis approach in the functional spatio-temporal model is not restricted to model the daily pattern of the data, and provides us more capability to explore the nature of the data of our interest.

## Figures and Tables

**Table 1:** The selection of model covariates according to AIC.

	Iteration									
**Season**	**Iter 0**	**Iter 1**	**Iter 2**	**Iter 3**	**Iter 4**	**Iter 5**	**Iter 6**	**Iter 7**	**Iter 8**	**Iter 9**
Spring	NO2	SO2	PM10	CO	PRES	UVB	Iws	IRAIN	TEMP	DEWP
Summer	NO2	PM10	SO2	TEMP	IRAIN	UVB	DEWP	PRES	CO	Iws
Autumn	NO2	SO2	TEMP	DEWP	PM10	UVB	CO	Iws	PRES	IRAIN

**Table 2:** Criteria $\bar{MSE}$, $\bar{R^{2}}$, and AIC under different numbers of Fourier basis.

$\phi {\left(h\right)}^{\prime }z\left(s,t\right)$	β(h)	$\sigma_{\varepsilon }^{2}$	$\bar{\mathrm{MSE}}$	$\bar{R^{2}}$	AIC
5	3	3	357.58	0.9206	−255,385
5	3	5	356.32	0.9209	−254,607
5	3	7	356.47	0.9208	−254,599
5	5	3	352.61	0.9215	−254,235
5	5	5	352.25	0.9216	−253,459
5	5	7	352.39	0.9215	−253,454
5	7	3	352.14	0.9215	−254,116
5	7	5	351.62	0.9217	−253,309
5	7	7	351.76	0.9217	−253,306
7	3	3	332.95	0.9259	−249,514
7	3	5	331.88	0.9261	−248,723
7	3	7	331.99	0.9261	−248,713
7	5	3	330.06	0.9264	−248,848
7	5	5	329.80	0.9264	−248,066
7	5	7	329.90	0.9264	−248,062
7	7	3	329.09	0.9266	−248,656
7	7	5	328.97	0.9266	−247,879
7	7	7	329.05	0.9266	−247,876
9	3	3	324.08	0.9278	−246,673
9	3	5	323.13	0.9280	−245,937
9	3	7	323.19	0.9280	−245,928
9	5	3	322.07	0.9281	−246,056
9	5	5	321.80	0.9282	−245,327
9	5	7	321.86	0.9282	−245,322
9	7	3	321.28	0.9283	−245,879
9	7	5	321.12	0.9283	−245,152
9	7	7	321.13	0.9283	−245,150

**Table 3:** $\bar{MSE}$, $\bar{R^{2}}$, and AIC for the five models.

	Number of Basis			Model Criteria				
	β	$\phi {\left(h\right)}^{\prime }z\left(s,t\right)$	$\sigma_{\epsilon }$	$\bar{\mathrm{MSE}}$	$\bar{R^{2}}$	**AIC**	**logL ^1^**	**Npar ^2^**
Equation (22)	0	0	0	1880.54	0.5863	−414,714	−414,700	7
Equation (23)	5	0	0	1171.55	0.7423	−395,874	−395,812	31
Equation (24)	0	0	0	552.7	0.879	−256,960	−256,940	10
Equation (25)	0	7	0	336.88	0.925	−252,426	−252,370	28
Equation (26)	5	7	0	329.8	0.9264	−248,066	−247,954	56

^1^ log likelihood, ^2^ number of parameters.

**Table 4:** $\chi^{2}$ tests for significance of fixed effects.

Covariate	$\chi^{2}$ Statistic	*p*-Value
Cons	282.77	0
$PM_{10}$	2114.06	0
$SO_{2}$	1048.50	0
$NO_{2}$	29,032.23	0
TEMP	5554.16	0
IRAIN	0.91	0.96
UVB	30,934	0

**Table 5:** Estimates and standard error of parameter g,θ, and v.

	Transition *g*		θ [km]		Variance *v*	
	Est	Std.err	Est	Std.err	Est	Std.err
Basis 1	0.739	0.018	63.12	4.57	8422.14	549.12
Basis 2	0.229	0.026	50.94	0.96	3799.47	176.33
Basis 3	0.179	0.03	36.98	1.02	2027.63	106.59
Basis 4	0.034	0.032	36.34	0.54	896.86	50.61
Basis 5	0.106	0.034	39.75	0.84	702.64	41.65
Basis 6	0.043	0.043	31.92	0.87	191.09	13.53
Basis 7	−0.210	0.042	37.10	0.35	151.80	10.78
